# Global Trends and Hotspots in Non-Targeted Screening of Water Pollution Research: Bibliometric and Visual Analysis

**DOI:** 10.3390/toxics12120844

**Published:** 2024-11-24

**Authors:** Yitian Wu, Yewen Shi, Tianmin Gu, Xiushuai Du, Zhiyuan Du, Chi Zhang, Ke Sun, Yue Zhang, Xiaojing Guo, Shenghan Wang, Weiwei Zheng, Yi He, Wei Liu

**Affiliations:** 1 Shanghai Municipal Center for Disease Control and Prevention, Shanghai 200336, China; wuyitian@scdc.sh.cn (Y.W.); shiyewen@scdc.sh.cn (Y.S.); gutianmin@scdc.sh.cn (T.G.); 2 Key Laboratory of the Public Health Safety, Ministry of Education, Department of Environmental Health, School of Public Health, Fudan University, Shanghai 200032, China; xsdu22@m.fudan.edu.cn (X.D.); 13035405580@163.com (Z.D.); 22301020109@m.fudan.edu.cn (Y.Z.); weiweizheng@fudan.edu.cn (W.Z.); 3 Key Laboratory of Health Technology Assessment, National Health Commission of the Peoples Republic of China, Fudan University, Shanghai 200032, China; czhang24@m.fudan.edu.cn; 4 School of Health Economics and Management, Nanjing University of Chinese Medicine, 138 Xianlin Road, Qixia District, Nanjing 210023, China; jojosun172@gmail.com; 5 KunShan Hospital of Traditional Chinese Medcine, Suzhou 215300, China; zyy_ywk@126.com; 6 School of Public Health, Nantong University, Nantong 226019, China; 2417110063@stmail.ntu.edu.cn; 7 Tongren Hospital, Shanghai Jiao Tong University School of Medicine, Shanghai 200336, China

**Keywords:** NTS, water pollution, bibliometrics, global trends, research hotspots

## Abstract

Non-targeted screening (NTS) technology has been showing significant potential in identifying contaminants of emerging concern (CECs) in water and has attracted great attention in academia in recent years. It is a method that analyzes samples without pre-selecting substances, enabling the detection and identification of unknown compounds, which is crucial for environmental health and public protection. This study uses the Bibliometrix package in R 4.4.1 and CiteSpace 6.3.R1 software to statistically analyze 589 relevant publications from the Web of Science Core Collection from 2007 to 2024. Our work concentrates on NTS of water bodies; thus, articles that only analyze water sediments without analyzing the water were not considered for inclusion. By conducting a quantitative analysis and visualizing the publication trends, countries, authors, journals, and keywords, the present study identifies research hotspots, compositions, and paradigms within this field, trying to analyze the horizontal and vertical development trends and structural evolution of the research area. The research found that the application of NTS in water pollution studies has progressed through three phases: theoretical exploration, rapid development, and steady progress. From the national level, China leads with the highest number of publications (131), followed by Germany (105), Spain (50), and the United States (39). The top three authors by publication volume are J. Hollender, Nikolaos S. Thomaidis, and Emma L. Schymanski, while the top three by citation count are J. Hollender, Emma L. Schymanski, and M. Krauss. However, international collaboration between countries and researchers still remains an area for improvement. Science of the Total Environment is the journal with the highest number of publications (81), and Environmental Science & Technology holds the highest number of citations. Research on NTS methodologies, suspect screening, and health risk assessments are hot topics in the academic community. Future research is expected to be multidisciplinary, with emerging hotspots likely to focus on including the identification of novel pollutants through NTS, toxicity assessments of biotransformed compounds, and the health impacts and mechanisms of related compounds.

## 1. Introduction

The quantity and types of chemicals released into the environment, as well as their impacts on public health, have long been focal points of research in the field of public health [1]. On 10 April 2024, the U.S. Environmental Protection Agency (EPA) released its first legally enforceable national drinking water standard, aimed at addressing the issue of forever chemicals, with limits for per- and polyfluoroalkyl substances (PFASs). The standard specifies allowable levels for six types of PFASs in drinking water. As the complexity of compounds entering water systems increases and research on emerging contaminants advances rapidly, effective water assessment tools and methods are becoming increasingly essential. While targeted screening for the assessment and routine monitoring of water is relatively well established and widely used in previous studies, this approach, which focuses on a small subset of known contaminants, plays an important role in detecting these substances. However, it may also result in the oversight of unknown or unexpected contaminants.

In recent years, high-resolution mass spectrometry (HRMS) has proven highly effective in accurately screening small-molecular-mass ranges and analyzing narrow chromatographic peaks. The combination of HRMS with soft ionization techniques, such as electrospray ionization, and liquid chromatography–high-resolution mass spectrometry (LC-HRMS) has significantly accelerated the development of non-targeted screening technologies (non-target screening, NTS) [2]. NTS is a comprehensive screening method for detecting unknown compounds in samples, as it does not depend on predefined target lists or standards. Sample processing methods typically involve broad-spectrum extraction techniques such as solid-phase extraction (SPE) or liquid–liquid extraction (LLE) to discover a wide range of analysts.

This study uses bibliometrics to perform a longitudinal and comprehensive analysis of research on non-targeted screening (NTS) for addressing water pollution and health-risk chemicals in water over the past 18 years (2007–2024). The objectives are as follows: (1) to present a comprehensive view of NTS in water pollution research by analyzing publication statistics from various countries/regions and institutions, and to foster collaboration and communication; (2) to offer a multidimensional evaluation of authors and journals, enabling researchers to quickly identify research directions, fields, and experts; (3) to clarify research hotspots, the current status, and development trends by analyzing keywords and highly cited studies, helping researchers pinpoint emerging research directions.

## 2. Methods

**Figure 1:**
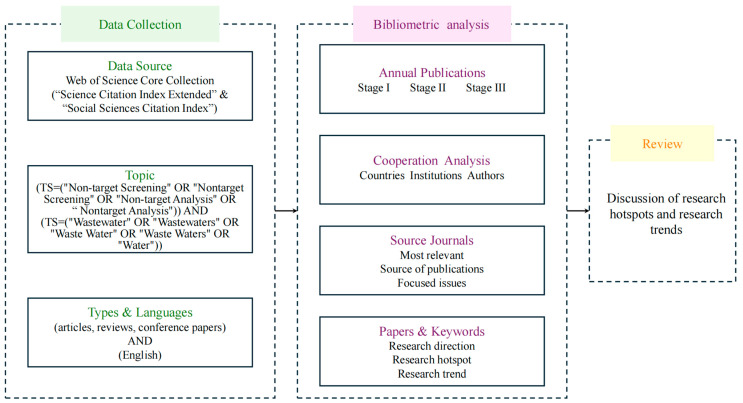
Schematic diagram of research process.

The data for this study were sourced from the Web of Science Core Collection (WOSCC) database, with search limits set to “Science Citation Index Expanded” and “Social Sciences Citation Index”. The WOSCC is a comprehensive database that includes high-quality journals across various disciplines and is the most commonly used database for bibliometric analysis internationally [3]. The search terms for NTS and water pollution research were as follows: (TS = (“Non-target Screening” OR “Nontarget Screening” OR “Non-target Analysis” OR “Nontarget Analysis”)) AND (TS = (“Wastewater” OR “Wastewaters” OR “Waste Water” OR “Waste Waters” OR “Water”)). The time span was set from 2007 to 2024, and the language was restricted to English, with document types including articles, reviews, and conference papers. Since the literature search date was conducted on 1 June 2024, the results for 2024 are considered as the output for half a year and included in the bibliometric analysis. A total of 744 search results were imported into Zotero (version 6.0), where they were manually checked for duplicates, incomplete studies, and those unrelated to the topic, resulting in 589 documents. These were downloaded in plain text format and used to establish the database. Bibliometric and visualization analyses were performed using the Bibliometrix package in R 4.4.1 and CiteSpace 6.3.R1 to provide a detailed analysis and summary of publication trends, countries, institutions, authors, source journals, key citations, and keywords, revealing the current state and dynamics of the field. The research flowchart is illustrated in Figure 1.

## 3. Results

### 3.1. Temporal Trends in Publication

**Figure 2:**
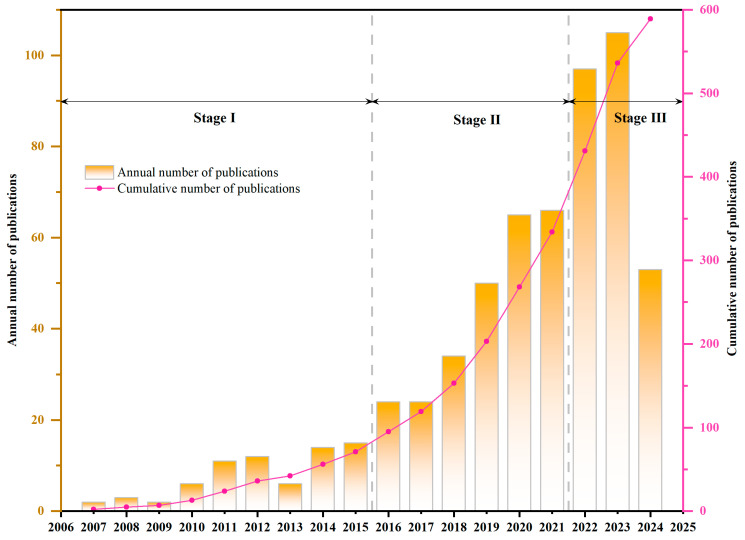
The temporal distribution of publications on NTS water pollution research from 2007 to 2023. The bar chart illustrates the annual number of publications with bars, while the red line depicts the cumulative total of publications over time.

As shown in Figure 2, the number of publications on NTS water pollution research has steadily increased from 2007 to 2023. This trend can be divided into three stages. **(1) Theoretical exploration stage (2007–2015)**. Due to technological limitations, research on water pollution during this period primarily focused on targeted screening of specific pollutants. NTS, as a new application of LC-MS in water contaminants, was in its early stages of theoretical exploration and application prospects [4]. During this time, research on NTS began to be applied to various fields such as industrial wastewater, natural water catchments, groundwater, and river sediments. The analytical framework for peak extraction, feature classification, and toxicity assessment, supported by databases, gradually took shape, with further advancements into relevant theories [5,6]. At the same time, there have been criticisms of NTS. Scholars like Nurmi, J pointed out with extensive practice that manually processing large volumes of NTS data often results in significant misidentification of compounds, making the process time-consuming and ineffective. They argued that improvements in analytical techniques were necessary for better application of NTS in environmental analysis using LC-MS systems [7]. Scholars such as Zedda, M have discussed the data entries contained in publicly available large MS chemical databases and expressed optimism for the role of composite digital libraries and computational tools in advancing the application of NTS [8]. **(2) Rapid development stage (2015–2021).** During this period, NTS feature-matching algorithms continued to improve. Public databases, such as Compound libraries [9], Online database mzCloud [10], The US EPA CompTox Chemistry Dashboard, NORMAN Database [11], The public databases of MS-DIAL [12], and MassBank Europe [13], along with numerous self-built databases, rapidly developed, providing technical and data resource support for NTS. In recent years, the number of articles on NTS research has grown steadily, increasing from 15 publications in 2015 to 97 in 2023, with an average annual growth of 12 articles. **(3) Steady development stage (2022–2024)**. Globally, the number of publications on NTS and water pollution has stabilized at over 100 per year. Research during this stage has expanded to cover agricultural water bodies, industrial waters, urban rivers, wastewater recycling, reservoirs, and drinking water [14,15]. Numerous previously overlooked contaminants have been identified, including PCBs, PFAS, pesticides, veterinary drugs, antibiotics, anticancer drugs, psychotropic drugs, microplastics, and their adsorbed pollutants, as well as transformation products of these chemicals, and suspected screening and toxicity analysis of these substances have been conducted [16]. The research trends across these three stages indicate that NTS of water bodies is becoming a hot research area and is expected to attract more attention in the future.

### 3.2. National/Regional and Institutional Analysis

Between 2007 and 2024, research on NTS water pollution spanned 60 countries/regions. Domestic interest in this field started later, with relatively few articles before 2016; however, research activity surged significantly afterward. As of 2024, at the national level, China leads with 131 papers, ranking first worldwide, followed by Germany (105), Spain (50), and the United States (39). However, from another perspective, the European Union has contributed a significant number of NTS manuscripts, positioning itself as a leader in advancing this field.

**Figure 3:**
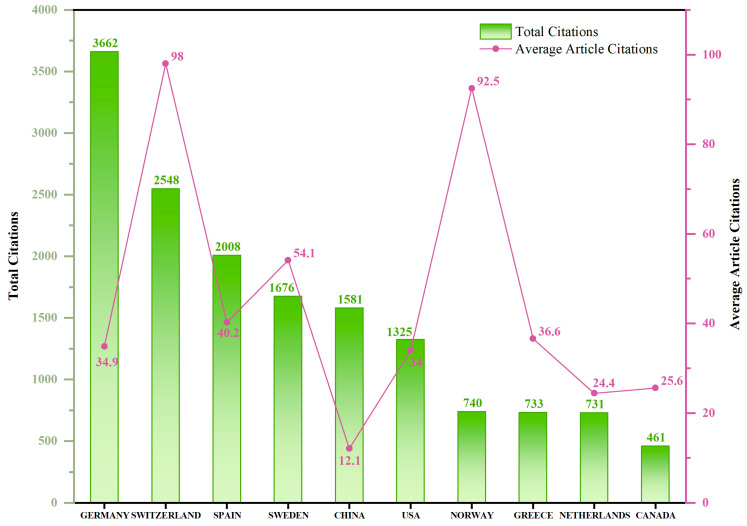
The distribution of total citations and average citations per paper by country. The bar chart illustrates the total citation count, while the line graph depicts the average number of citations per article.

The number of citations reflects the academic impact of the papers [17]. Switzerland has the highest average citation count at 98, indicating the high average impact of its research results. Germany holds the highest total citation count, with 3662 citations and an average of 35 citations per paper. China, despite having the largest number of published papers, has a total of 1581 citations, with an average of only 12 citations per paper (Figure 3). This suggests that although China leads in publication volume, the quality of its literature varies, and its international research impact could be further improved.

**Figure 4:**
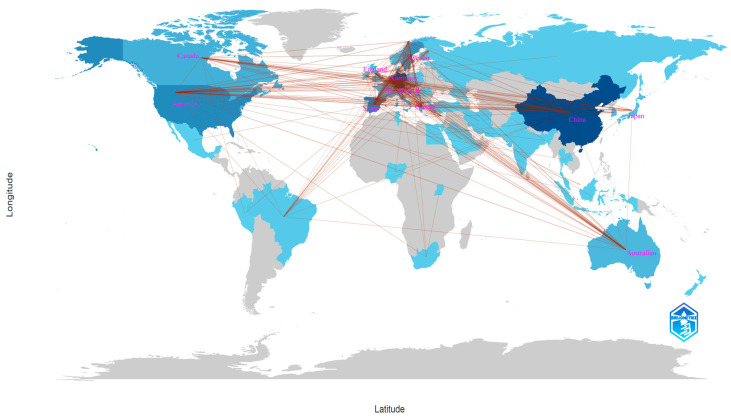
A national collaboration map based on statistics of all authors’ affiliated countries. The connections between countries/regions are indicated by lines.

Figure 4 visualizes the national collaboration map, where darker colors indicate higher publication volumes and denser lines represent closer cooperation. Germany has the most international collaborations, followed by Spain, the United States, Sweden, China, and the Netherlands. Single-country publications (SCPs) and multiple-country publications (MCPs) are often used to measure contributions to international cooperation. Supplementary Table S1 shows the number of papers published by each country, as well as their SCP, MCP, and MCP ratios. While China, Germany, Spain, and the United States are major contributors to this field, their MCP ratios remain relatively low (0.16, 0.25, 0.26, and 0.28, respectively). This indicates that the field of NTS water pollution research still lacks sufficient global collaboration. Future efforts should focus on strengthening international partnerships across different geographic regions to advance the field.

**Figure 5:**
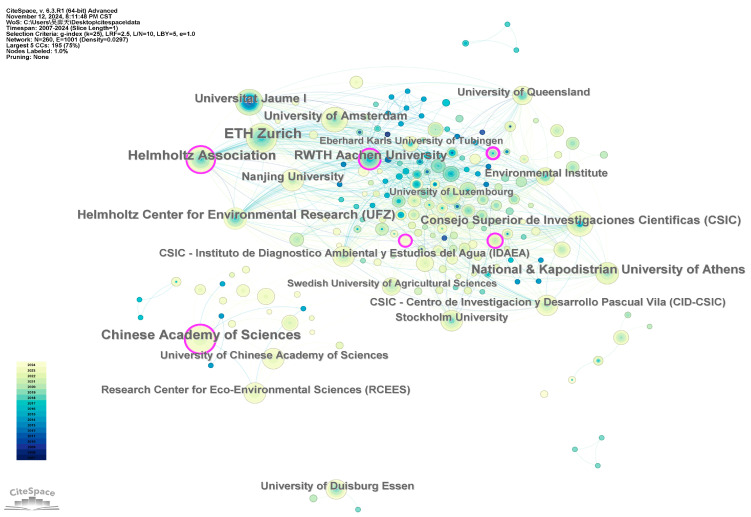
Analysis of collaborative network visualization of institutions. The nodes of different colors represent the institutions of different clusters in different years, the size of the nodes indicates the frequency of their occurrence, the lines represent collaborative relationships and the purple outer ring indicates that the node has high betweenness centrality.

Figure 5 illustrates the institutional collaboration network. In the map, the nodes represent institutions, with larger nodes indicating a higher number of published papers and closer relationships with other institutions. The connections between nodes represent co-occurrence relationships, with line thickness indicating the strength of co-occurrence.

The top 10 institutions by publication volume are mainly from Germany, Switzerland, and China. These include ETH Zurich (48), followed by the Chinese Academy of Sciences (40), the Helmholtz Association (40), the Helmholtz Center for Environmental Research (UFZ) (33), the National & Kapodistrian University of Athens (31), RWTH Aachen University (29), Universitat Jaume I (25), Consejo Superior de Investigaciones Cientificas (CSIC) (24), the University of Amsterdam (24), and Nanjing University (21), as shown in Supplementary Table S2.

Centrality is a commonly used measure to gauge the importance of nodes in network mapping in CiteSpace, indicating the frequency of collaboration and communication with other institutions. The Department of Environment and Climate Change Canada ranks highest in centrality (0.21), followed by Zhejiang University (0.18), the Chinese Academy of Sciences (0.13), RWTH Aachen University (0.12), the Centre National de la Recherche Scientifique (CNRS) (0.11), the Helmholtz Association (0.10), the University of Queensland (0.10), Nanjing University (0.09), ETH Zurich (0.07), and the Helmholtz Center for Environmental Research (UFZ) (0.07). These institutions are considered core nodes in the network, signifying a greater impact in the field (Supplementary Table S3). The analysis of the institutional collaboration network reveals that institutions with higher publication volumes generally have broader collaboration networks.

### 3.3. Author Analysis

**Figure 6:**
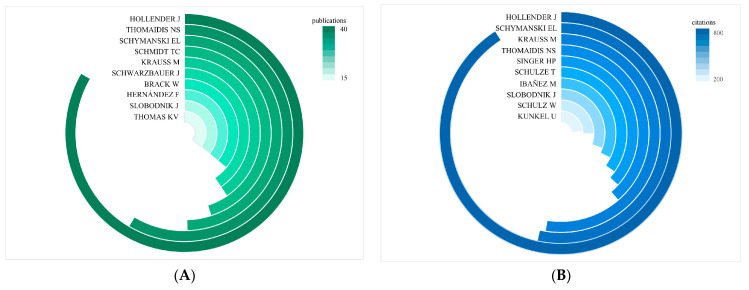

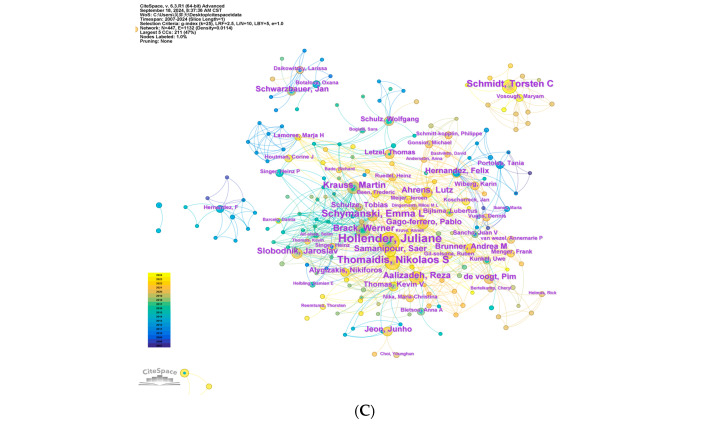
(**A**) The top 10 authors by number of publications. (**B**) The top 10 authors by number of citations. (**C**) Author collaboration network. Nodes of varying hues signify authors who collaborated in distinct years, while the dimensions of these nodes reflect the frequency of their appearance.

Between 2007 and 2024, a total of 444 authors contributed to research on NTS and water pollution. The distribution of authorship in this field follows Lotka’s law [18], with over 90% of the authors (407 individuals) contributing fewer than five papers. J. Hollender from the Swiss Federal Institute of Aquatic Science and Technology is the most prolific author with 37 publications, followed by Nikolaos S. Thomaidis from the National and Kapodistrian University of Athens with 26 papers, and Emma L. Schymanski from the Luxembourg Centre for Systems Biomedicine with 22 papers (Figure 6A). In terms of citations, J. Hollender leads with 774 citations, followed by Emma L. Schymanski with 455 citations, M. Krauss from the Helmholtz Centre for Environmental Research with 447 citations, and Nikolaos S. Thomaidis with 330 citations (Figure 6B).

The H-index, a widely used hybrid quantitative measure, evaluates both the quantity and impact of a researcher’s academic output [19,20]. Our analysis identified four authors with notable contributions to the field of NTS and water pollution, each possessing a high H-index: J. Hollender (70), Nikolaos S. Thomaidis (69), M. Krauss (52), and Emma L. Schymanski (45).

J. Hollender’s research focuses on natural water bodies such as oceans, rivers, and sediments, investigating the sources of pollutants and the impact of wastewater from industrial and agricultural activities, as well as current wastewater treatment methods, on the types, quantities, and distribution of pollutants [21,22]. In recent years, there has been increasing academic focus on the toxic risks of complex mixtures, unknown chemicals, and new pollutants. Hence, this researcher’s focus has also gradually shifted toward new pollutants [23,24]. Nikolaos S. Thomaidis is known for his innovations in methodology. He has proposed compound calculation methods based on non-targeted temporal models and explored retention time indices for newly emerging pollutants using quantitative structure–retention relationship models. These advancements provide critical methodological support for NTS [25,26]. Additionally, he has explored effective computational frameworks for deep learning traditional neural networks and novel analytical methods such as reversed-phase ultra-high-performance liquid chromatography–electrospray quadrupole time-of-flight tandem mass spectrometry. These techniques have been applied in practical studies of urban water pollution [27]. Emma L. Schymanski and Nikolaos S. Thomaidis have a collaborative relationship. They have built on methodological theory and applied research, proposing an extension to the original NORMAN prioritization scheme to include strategies for chemicals beyond the targeted ones [28].

Analysis of the author collaboration network reveals that high-level researchers in this field have formed various research teams with distinct focuses, and high-productivity authors frequently collaborate with one another (Figure 6C). This analysis reveals that the aforementioned individuals, along with other researchers who have conducted extensive studies in the field of NTS, are often closely associated with the NORMAN Association and frequently collaborate with each other. This organization specializes in NTS work and conducts numerous collaborative experiments annually, contributing significantly to the EU’s leading position in this field; moreover, some senior researchers mentor top students in the field and often serve as the first authors of scientific publications, further advancing research efforts. To advance NTS water pollution research, it is recommended to further enhance communication and collaboration among scholars from different countries and with varying research focuses.

### 3.4. Journal Source Analysis

Between 2007 and 2024, a total of 97 academic journals published research papers related to NTS and water pollution. The journal with the highest number of publications is Science of the Total Environment, with 81 papers, accounting for 13.61% of the total. It is followed by Environmental Science & Technology (76 papers, 12.77%), Water Research (50 papers, 8.40%), Journal of Hazardous Materials (35 papers, 5.88%), and Analytical and Bioanalytical Chemistry (35 papers, 5.88%) (Supplementary Table S4).

**Figure 7:**
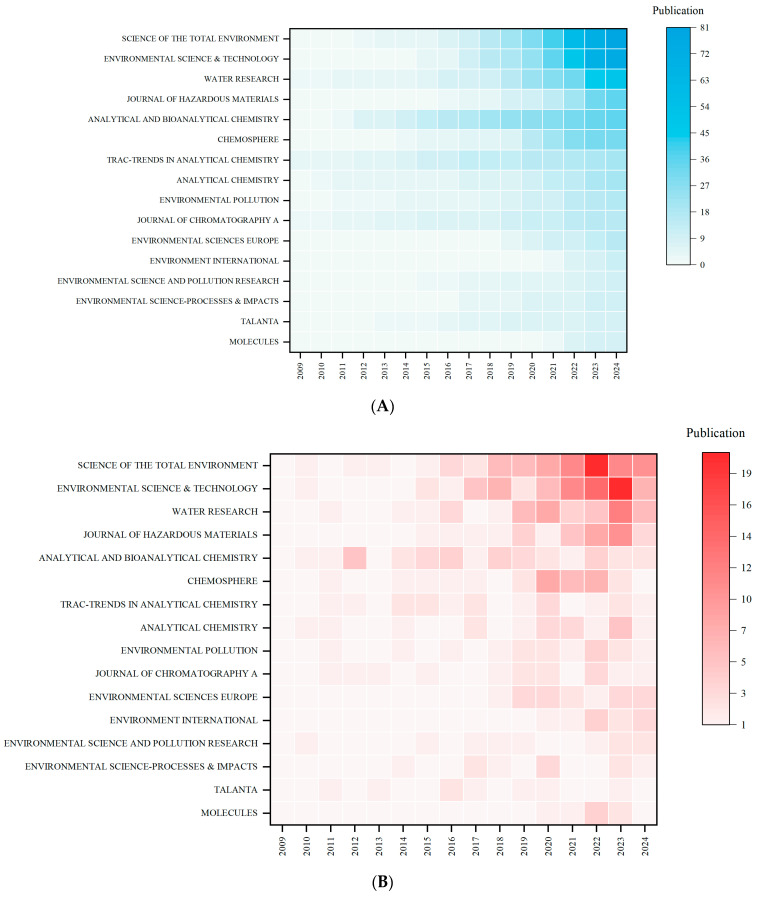

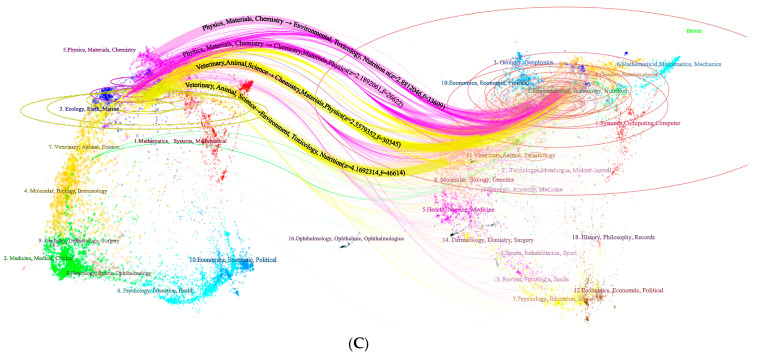
(**A**) The number of journal articles over time, with darker colors indicating a higher number of publications. (**B**) The number of journal articles increases over time, with darker colors indicating a higher volume of publications. (**C**) The dual-map overlay of journals. Citing journals are on the left, cited journals are on the right, the lines represent the flow of information, and the colored paths indicate citation relationships.

Over the past three and a half years, the annual publication growth for Science of the Total Environment, Environmental Science & Technology, Water Research, Journal of Hazardous Materials, and Analytical and Bioanalytical Chemistry has been 15.5, 14.8, 8.3, 7.8, and 2.8 papers, respectively (Figure 7A,B), indicating a sharp rise in the attention these journals are giving to the field. Among them, Environmental Science & Technology is the most influential, with the highest citation count of 5003. It is followed by Science of the Total Environment (2214 citations), Water Research (2020 citations), Chemosphere (1464 citations), Analytical Chemistry (1373 citations), and Journal of Chromatography A (1351 citations).

The journal co-citation network, constructed through a dual-network overlay, visually reflects the flow of disciplinary knowledge and research trends [29]. In Figure 7C, the right side shows the distribution of cited journals, while the left side represents the citing journals. The flow of disciplinary knowledge moves from right to left, forming a stream of information. Cited journals provide the theoretical and technical foundation for related research, while citing journals represent the hotspots and trends in the field. The information flow visualizes the development process and evolutionary direction of the discipline, with convergence points indicating key research areas and trends [30].

The fields of “environmental science, toxicology, nutrition” “chemistry, materials science, physics”, and “botany, ecology, geography, geology, geophysics” serve as the primary theoretical foundations for research on NTS and water pollution. Recent studies have predominantly focused on “environmental science, toxicology, nutrition” and “chemistry, materials science, physics”, with significant citations apperaing in journals related to “physics, materials science, chemistry” and “veterinary science, zoology, science”. This aligns with earlier findings, highlighting continued growth in areas such as livestock, agriculture, chemical engineering, materials science, and environmental and water pollution issues, which are gradually becoming hot topics for future research. The presence of multiple substantial information flows suggests a rising number of interdisciplinary studies, with the integration of cross-disciplinary knowledge being a key direction for future advancements.

### 3.5. Keyword Analysis

#### 3.5.1. Keyword Frequency and Clustering

**Figure 8:**
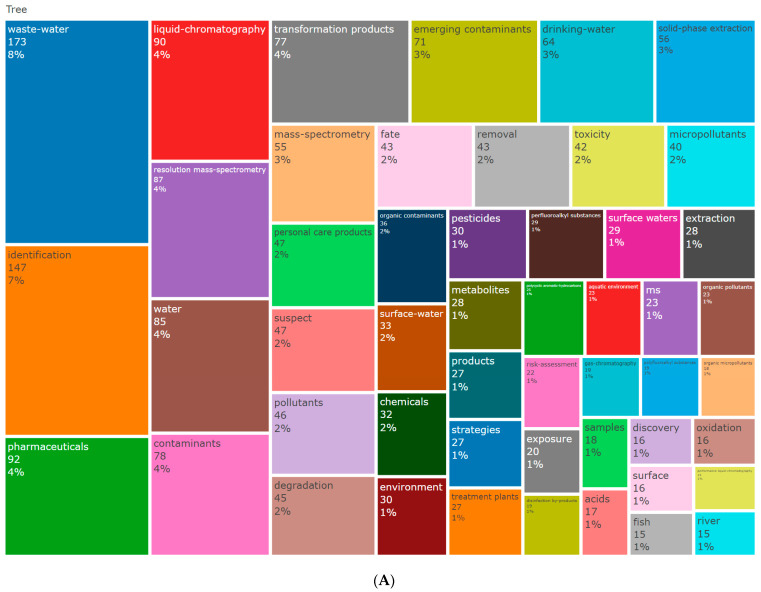

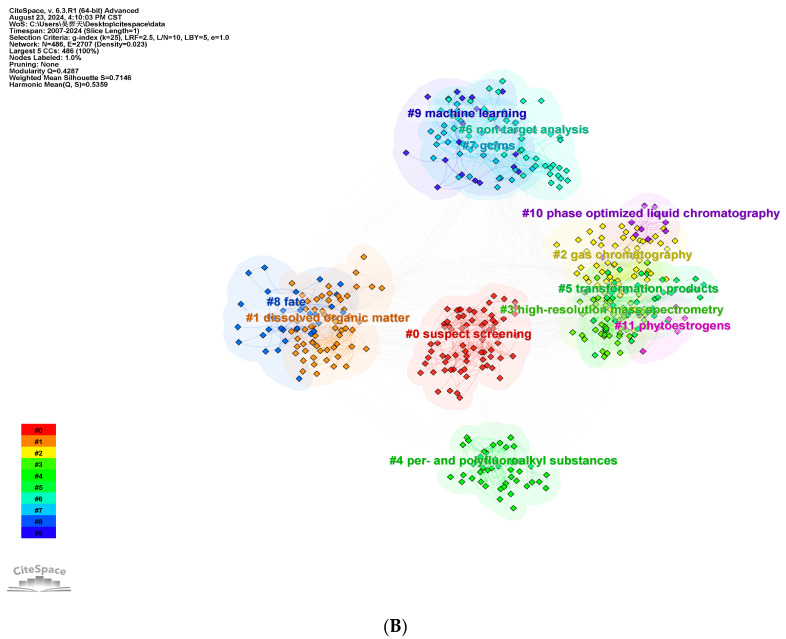
(**A**) Keyword dendrogram. The size of the grid represents the frequency of keyword appearance. (**B**) Keyword clustering. The nodes in different colors represent the keywords in different clusters, and the size of the nodes indicates the frequency of their occurrence.

Keywords serve as high-level summaries of research content. Analyzing the frequency and co-occurrence of keywords in the scientific literature can reveal core research themes and help identify important topics and trends in a specific field [31]. Figure 8A displays a keyword dendrogram, where the numbers in the color blocks represent the frequency of keyword occurrences and the percentages indicate the significance of keywords relative to the entire keyword set (based on term frequency–inverse document frequency). From 2007 to 2023, publications related to NTS and water pollution included 3735 keywords provided by authors. The five most frequently used keywords are “water pollution” (173 times), “identification” (147 times), “drugs” (92 times), “liquid chromatography” (90 times), and “high-resolution mass spectrometry” (87 times). “Emerging pollutants”, which appeared 71 times, is the most frequent keyword in environmental media categories. In terms of toxicity and mechanisms, “toxicity” and “risk assessment” are the most commonly used keywords.

Using CiteSpace, keywords were clustered with the log-likelihood ratio (LLR) algorithm to extract keyword labels, as shown in Figure 8B [32]. In the clustering diagram, modularity and silhouette are important parameters for evaluating clustering effectiveness. These values range from 0 to 1, with values closer to 1 indicating tighter connections between nodes within clusters and better clustering results. A modularity value >0.3 signifies a significant clustering network structure, while a weighted average silhouette value >0.7 indicates high confidence in the results [33]. In this study, the modularity value and weighted average silhouette value were 0.4287 and 0.7146, respectively, indicating that the clustering structure is well defined and uniform, with a high level of confidence in the clustering results. Clusters are numbered from 0, with smaller numbers indicating larger clusters. The major categories with more than 20 members were selected for analysis. Between 2007 and 2023, 11 major research directions were identified. The largest cluster is “suspected target screening”, followed by “soluble organic compounds”, “gas chromatography”, “high-resolution mass spectrometry”, and “alkyl and polyfluoroalkyl substances”.

The clustering results indicate that from 2007 to 2024, research on NTS water pollution has primarily focused on three areas:

(1) Advances in NTS and related analytical methods: This includes updates and iterations of gas chromatography-based methods [34], liquid chromatography-based methods [35], high-resolution mass spectrometry [36], and machine learning techniques [37]. These methods are used to assess the temporal and spatial distribution of compounds across various water-related environmental media (natural water, groundwater, urban industrial wastewater, agricultural irrigation channels, and tap water) and their exposure burden on organisms.
(2) Comprehensive identification of water body compounds: NTS is employed to broadly characterize waterborne compounds, establish databases, and combine suspect and target screening methods for comparison. Research in this area focuses on single and polyalkyl substances, soluble organic compounds, plant estrogens, and their transformation products.
(3) Health impacts of waterborne compounds: This research primarily evaluates pollutants through hazard characterization assessments and in vitro bioassays, focusing on the environmental persistence, bioaccumulation, in vivo toxicity, and in vitro toxicity of pollutants. Future environmental monitoring and regulation should take these pollutants into thorough consideration [38].

#### 3.5.2. Keyword Trend Analysis

To track evolving research trends from 2007 to 2023, an analysis of keyword burst intensity was conducted, identifying 25 keywords with high burst intensity, as shown in Table 1, the color bars in the table represent the temporal burstiness of keywords, with red indicating a sharp increase in the number of citations for a keyword within a specific time period, showing rapid changes in research hotspots. The top five keywords by burst intensity are “pollutants” (6.11), “non-targeted screening” (4.94), “identification” (4.71), “organic micropollutants” (4.36), and “effect-oriented analysis” (4.16). The keywords with burst intensity extending into 2024 include “non-targeted screening”, “perfluorinated substances”, “reaction kinetics”, “disinfection by-products”, and “biotransformation” [39].

**Figure 9:**
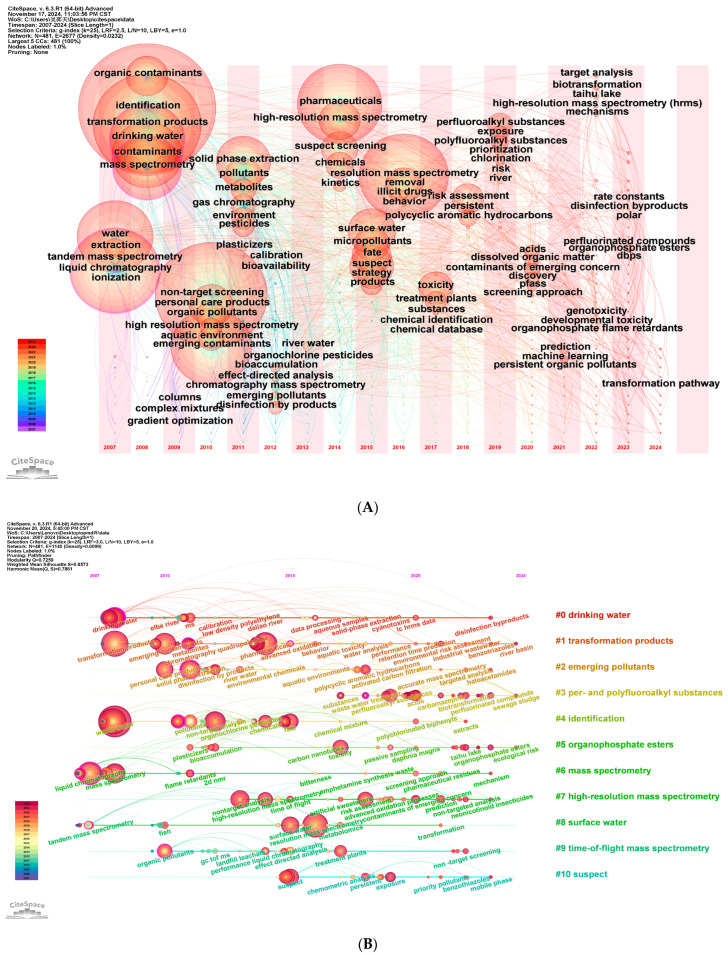
(**A**) A thematic pathway graph. Each circle in the graph represents a keyword, with the position of the circle indicating the year when the keyword first appeared. The purple outer ring indicates that the node has high betweenness centrality. (**B**) A keyword timeline graph. This graph represents the development of keywords within a specific keyword cluster over different years, reflecting changes in research trends.

Figure 9A depicts a timeline graph showing the evolution of keywords over time. Each circle represents a keyword, with its position indicating the year it first appeared and its size reflecting the frequency of occurrence. It can be observed that a substantial number of keywords related to NTS in water pollution research emerged before 2015. The size of the circles suggests that these keywords have remained central to NTS water pollution research throughout the study period. Figure 9B illustrates the development trends of various topics within keyword clusters over different years. It is a further subdivision of the keywords over time after clustering, allowing us to understand the changes in research trends by examining the development trajectory of keywords within each cluster. The two figures demonstrate that terms such as “large volume injection”, “new contaminants”, “perfluorinated compounds”, “biotransformation”, “dissolved organic matter”, “prioritization”, “prediction”, and “machine learning” have emerged more recently and have become quite popular in recent years.

Overall, during the theoretical exploration phase (2007–2015), the understanding of NTS technology was still in its infancy. Research primarily focused on establishing and refining analytical techniques such as gas chromatography, liquid chromatography, and solid-phase extraction [40,41]. The rapid development phase (2015–2021) witnessed the widespread use of NTS for the broad identification of compounds in various water environments, bringing a significant number of unknown pollutants into the research spotlight. During this period, animal experiments with model organisms like zebrafish and mice significantly advanced studies on the health hazards posed by pollutants [42,43,44]. The steady development phase (2022–2024) saw advancements in mass spectrometry and breakthroughs in algorithms, particularly in machine learning, which further improved exposure analysis [44]. NTS has begun to be applied in previously overlooked regions, such as parts of Africa, with a renewed focus on emerging pollutants, microplastics, perfluorinated substances, and biotransformation compounds [45].

Looking ahead, there is an urgent need to address key challenges such as toxicity testing, population exposure assessment, metabolic pattern analysis, and the safety evaluation of compounds.

## 4. Discussion and Conclusions

This study included a total of 589 relevant articles, exploring the current status, research hotspots, and future trends of NTS water pollution research from 2007 to 2024. The findings are summarized as follows:

(1) Phases of Research Development: From 2007 to 2024, NTS water pollution research can be categorized into three phases. Since 2015, the research has entered a rapid development phase, with an annual increase of 12 publications. China leads in the number of publications, but its average citation per paper is relatively low, at only 12. Global collaboration in this field remains limited, as reflected by the MCP ratios across countries. The Swiss Federal Institute of Technology and the Chinese Academy of Sciences are leading institutions in this field. Based on a comprehensive evaluation, Hollender, J and Thomaidis, Nikolaos S emerge as the most influential researchers in this field.
(2) Journal Contributions: Between 2007 and 2024, papers related to NTS water pollution research were published in 97 different academic journals. Most research in this field is published in high-impact journals, with the highest number of papers published in “Science of the Total Environment”. Based on various impact assessment metrics, “Environmental Science & Technology” is the most influential journal in this field. Cluster analysis indicates that future journal research on NTS water pollution will focus on interdisciplinary fields such as livestock, agriculture, chemical engineering, materials, and environmental ecology.
(3) Shifts in Research Focus: Keyword analysis reveals that from 2007 to 2024, the research focus has gradually shifted from technical exploration of NTS to the broad identification of compounds in water environments and in-depth studies of their toxic effects. Current research is mainly centered on exposure risk assessment and health hazard mechanisms of emerging pollutants, microplastics, perfluorinated substances, and biotransformation compounds. Future research is expected to prioritize toxicity evaluation, population exposure assessment, metabolic pattern analysis, and safety evaluations of compounds and their impacts on public health.

As an emerging analytical technique, NTS has demonstrated significant potential in the monitoring of environmental and food-related chemical hazards due to its capacity to identify a vast array of compounds [46]. This study focuses on the field of water bodies, visualizing the trends, development, and potential of existing NTS water pollution research. However, NTS is not without limitations at its current stage of development. First, NTS technology lacks standardized sample pretreatment processes and evaluation criteria, making it challenging to standardize or reliably assess the results of its analyses [47]. Second, NTS can only provide the molecular formula and structural information of compounds, without determining their exact names and properties, thus requiring further experiments and analyses to verify the authenticity and toxicity of potential new pollutant candidates [48,49]. Third, NTS technology relies heavily on standard databases for compound identification [50]. The current lack of comprehensive and standardized high-resolution mass spectrometry (HRMS) databases for chemical hazards hampers the complete identification of unknown chemical hazards in complex samples. This also affects the comparability of compound identification based on different database matches [51]. Finally, the data analysis software and the algorithms for spectrum data analysis that NTS technology relies on vary, and the high learning threshold limits the widespread application of NTS technology. These challenges underscore the need for ongoing advancements in NTS technology. Improvements in standardization, database development, and user-friendly analytical tools will be critical to maximizing its value and applicability in environmental monitoring and management.

Although bibliometric analysis provides a macro, quantitative, and objective view of research hotspots and development trends, this study has some limitations. Firstly, it only used WOSCC data. While the WOSCC includes most high-quality research papers and generally does not significantly impact overall trends, omissions from databases like Scopus and PubMed may introduce bias. Secondly, this study only included research papers and reviews published in English, which may lead to language bias and data omission. Future research could utilize mixed databases without language restrictions for a more comprehensive bibliometric analysis of NTS water pollution research.

## Figures and Tables

**Table 1:** Keyword burst intensity table.

Keywords	Strength	Begin	End	2007–2023
accurate mass	4.08	2007	2015	▃▃▃▃▃▃▃▃▃ ▂▂▂▂▂▂▂▂▂
triple quadrupole	4.04	2007	2016	▃▃▃▃▃▃▃▃▃▃ ▂▂▂▂▂▂▂▂
samples	3.23	2007	2015	▃▃▃▃▃▃▃▃▃ ▂▂▂▂▂▂▂▂▂
confirmation	3.19	2007	2011	▃▃▃▃▃ ▂▂▂▂▂▂▂▂▂▂▂▂▂
organic contaminants	3.28	2008	2014	▂ ▃▃▃▃▃▃▃ ▂▂▂▂▂▂▂▂▂▂
quantification	3.3	2010	2011	▂▂▂ ▃▃ ▂▂▂▂▂▂▂▂▂▂▂▂▂
organic pollutants	2.52	2010	2014	▂▂▂ ▃▃▃▃▃ ▂▂▂▂▂▂▂▂▂▂
pollutants	6.11	2009	2016	▂▂ ▂▂ ▃▃▃▃▃▃ ▂▂▂▂▂▂▂▂
Solid-phase extraction	2.8	2011	2012	▂▂▂▂ ▃▃ ▂▂▂▂▂▂▂▂▂▂▂▂
chromatography mass spectrometry	3.02	2012	2019	▂▂▂▂▂ ▃▃▃▃▃▃▃▃ ▂▂▂▂▂
Gc/tofms	2.63	2012	2013	▂▂▂▂▂ ▃▃ ▂▂▂▂▂▂▂▂▂▂▂
time-of-flight mass spectrometry	2.63	2012	2013	▂▂▂▂▂ ▃▃ ▂▂▂▂▂▂▂▂▂▂▂
identification	4.71	2008	2017	▂ ▂▂▂▂▂▂ ▃▃▃▃ ▂▂▂▂▂▂▂
waste water	3.21	2008	2017	▂ ▂▂▂▂▂▂▂ ▃▃▃ ▂▂▂▂▂▂▂
organic micropollutants	4.36	2008	2021	▂ ▂▂▂▂▂▂▂▂ ▃▃▃▃▃▃ ▂▂▂
effect directed analysis	4.16	2015	2020	▂▂▂▂▂▂▂▂ ▂▂ ▃▃▃▃ ▂▂▂▂
in vitro	3.07	2018	2020	▂▂▂▂▂▂▂▂▂▂▂ ▃▃▃ ▂▂▂▂
emerging pollutants	2.63	2012	2019	▂▂▂▂▂ ▂▂▂▂▂▂ ▃▃ ▂▂▂▂▂
river	2.76	2019	2021	▂▂▂▂▂▂▂▂▂▂▂▂ ▃▃▃ ▂▂▂
non-target screening	4.94	2014	2024	▂▂▂▂▂▂▂ ▂▂▂▂▂▂▂▂ ▃▃▃
perfluoroalkyl substances	3.91	2019	2024	▂▂▂▂▂▂▂▂▂▂▂▂ ▂▂▂ ▃▃▃
kinetics	3.51	2014	2024	▂▂▂▂▂▂▂ ▂▂▂▂▂▂▂▂ ▃▃▃
non-target analysis	2.56	2013	2024	▂▂▂▂▂▂ ▂▂▂▂▂▂▂▂▂ ▃▃▃
disinfection by-products	2.47	2012	2024	▂▂▂▂▂ ▂▂▂▂▂▂▂▂▂▂ ▃▃▃
biotransformation	2.45	2022	2024	▂▂▂▂▂▂▂▂▂▂▂▂▂▂▂ ▃▃▃

## Supplementary Materials

The following supporting information can be downloaded at: https://www.mdpi.com/article/10.3390/toxics12120844/s1, Supplementary Table S1: Overview of Publication Numbers, SCP, MCPs, and MCP Ratios by Country; Supplementary Table S2: Overview of the number of publications by different institutions; Supplementary Table S3: Display of centrality among different institutions; Supplementary Table S4: Total number of publications by different journals in the field of non-targeted water pollution.
