# Supplementary material for: Global Trends and Hotspots in Non-Targeted Screening of Water Pollution Research: Bibliometric and Visual Analysis
Source: Toxics. 2024 Nov 24;12(12):844. doi: 10.3390/toxics12120844 (PMC11679217; doi:10.3390/toxics12120844)
Supplement: Supplementary file 1 [file toxics-12-00844-s001.zip › Supplementary table S1.pdf]

Supplementary Table S1: Overview of Publication Numbers, SCP, MCPs, and MCP Ratios by Country

Country	Articles	Articles %	SCP	MCP	MCP %
CHINA	131	22	110	21	16
GERMANY	105	17.6	79	26	24.8
SPAIN	50	8.4	37	13	26
USA	39	6.6	28	11	28.2
SWEDEN	31	5.2	19	12	38.7
NETHERLANDS	30	5	16	14	46.7
SWITZERLAND	26	4.4	16	10	38.5
GREECE	20	3.4	13	7	35
KOREA	20	3.4	17	3	15
CANADA	18	3	16	2	11.1
DENMARK	15	2.5	12	3	20
ITALY	13	2.2	7	6	46.2
AUSTRALIA	12	2	1	11	91.7
FRANCE	12	2	8	4	33.3
JAPAN	8	1.3	4	4	50
NORWAY	8	1.3	4	4	50
CZECH REPUBLIC	5	0.8	3	2	40
RUSSIA	5	0.8	4	1	20
UNITED KINGDOM	5	0.8	3	2	40
BRAZIL	4	0.7	2	2	50
FINLAND	4	0.7	4	0	0
INDIA	4	0.7	4	0	0
POLAND	4	0.7	4	0	0
BELGIUM	3	0.5	1	2	66.7
IRAN	3	0.5	1	2	66.7
LUXEMBOURG	3	0.5	0	3	100
AUSTRIA	2	0.3	0	2	100
SOUTH AFRICA	2	0.3	1	1	50
THAILAND	2	0.3	0	2	100
TURKEY	2	0.3	2	0	0
CROATIA	1	0.2	1	0	0
MEXICO	1	0.2	0	1	100
NEW ZEALAND	1	0.2	1	0	0
PERU	1	0.2	0	1	100
ROMANIA	1	0.2	1	0	0
SLOVAKIA	1	0.2	0	1	100
TUNISIA	1	0.2	0	1	100
