# Supplementary material for: Global Trends and Hotspots in Non-Targeted Screening of Water Pollution Research: Bibliometric and Visual Analysis
Source: Toxics. 2024 Nov 24;12(12):844. doi: 10.3390/toxics12120844 (PMC11679217; doi:10.3390/toxics12120844)
Supplement: Supplementary file 1 [file toxics-12-00844-s001.zip › Supplementary table S2.pdf]

Supplementary Table S2: Overview of the number of publications by different institutions

Affiliation	Articles
ETH Zurich	48
Chinese Academy of Sciences	40
Helmholtz Association	40
Helmholtz Center for Environmental Research (UFZ)	33
National & Kapodistrian University of Athens	31
RWTH Aachen University	29
Universitat Jaume I	25
Consejo Superior de Investigaciones Cientificas (CSIC)	24
University of Amsterdam	24
Nanjing University	21
CSIC - Instituto de Diagnostico Ambiental y Estudios del Agua (IDAEA)	20
Stockholm University	20
CSIC - Centro de Investigacion y Desarrollo Pascual Vila (CID-CSIC)	20
University of Chinese Academy of Sciences	20
Research Center for Eco-Environmental Sciences (RCEES)	19
University of Duisburg Essen	19
University of Queensland	18
Environmental Institute	17
Eberhard Karls University of Tubingen	14
University of Luxembourg	14
Swedish University of Agricultural Sciences	14
Norwegian Institute for Water Research (NIVA)	13
Changwon National University	13
University of Copenhagen	12
Technical University of Munich	11
Guangzhou Institute of Geochemistry	11
Vrije Universiteit Amsterdam	11
Centre National de la Recherche Scientifique (CNRS)	10
Institut Catala de Recerca de l'Aigua (ICRA)	10
South China Normal University	10
Orebro University	9
KWR Watercycle Research Institute	9
INRAE	8
Aarhus University	8
Utrecht University	8
Nankai University	7
Chinese Research Academy of Environmental Sciences	6
Consiglio Nazionale delle Ricerche (CNR)	6
Masaryk University Brno	6
NILU	6
University of Washington	6
University of Washington Tacoma	6

State University of New York (SUNY) System	6
Universitat de Girona	6
IVL Swedish Environmental Research Institute	5
Universidad de Almeria	5
Bureau de Recherches Geologiques et Minieres (BRGM)	5
Cornell University	5
Dongguan University of Technology	5
Aristotle University of Thessaloniki	5
Universidade de Santiago de Compostela	5
University of Antwerp	5
Helmholtz-Center Munich - German Research Center for Environmental Health	5
Environment & Climate Change Canada	4
Zhejiang University	4
Colorado School of Mines	4
Tongji University	4
BOKU University	4
Chinese Academy of Agricultural Sciences	4
Institut National de l'Environnement Industriel et des Risques (INERIS)	4
Universite Paris Cite	4
Umea University	4
Agilent Technologies	4
McGill University	4
University of Maryland Center for Environmental Science	4
Goethe University Frankfurt	4
University System of Maryland	4
Leipzig University	4
University of Helsinki	4
University of Basque Country	4
National Institute of Environmental Research (NIER)	4
Wageningen University & Research	4
Fed Inst Hydrol	4
Peking University	3
South China University of Technology	3
Norwegian University of Science & Technology (NTNU)	3
Ecole Polytechnique Federale de Lausanne	3
Hainan University	3
CIBERESP	3
Leuphana University Luneburg	3
Nanjing University of Science & Technology	3
CIBER - Centro de Investigacion Biomedica en Red	3
Ghent University	3
Korea University	3
Guangdong University of Technology	3
Chongqing University	3

China University of Geosciences	2
Jinan University	2
Leibniz Institut fur Pflanzenbiochemie	2
United States Environmental Protection Agency	2
Universidade Estadual de Campinas	2
Slovak University of Technology Bratislava	2
EC JRC ISPRA Site	2
Hsch Fresenius Gem GmbH	2
IMT Mines Ales	2
IMT - Institut Mines-Telecom	2
Free University of Berlin	2
European Commission Joint Research Centre	2
University of Toronto	2
Universite de Montreal	2
VITO	2
CNRS - Institute of Chemistry (INC)	2
Clemson University	2
Baylor University	2
Ecole des Ponts ParisTech	2
University of Milan	2
University of Georgia	2
Istituto Superiore di Sanita (ISS)	2
University of California System	2
CSIC - Instituto de Acuicultura de Torre de la Sal (IATS)	2
University of Washington Seattle	2
Agriculture & Agri Food Canada	2
Cochin University Science & Technology	2
Universite de Montpellier	2
Federal Institute for Materials Research & Testing	2
Chalmers University of Technology	2
Universite Paris Saclay	2
Minist Ecol & Environm	2
Hanyang University	2
Fraunhofer Gesellschaft	2
Istituto di Ricerca sulle Acque (IRSA-CNR)	2
Lanxess	2
Konya Technical University	2
Chungnam National University	2
Aix-Marseille Universite	2
University of Padua	2
Thermo Fisher Scientific	2
Ministry of Marine Affairs and Fisheries	2
Universite Paris-Est-Creteil-Val-de-Marne (UPEC)	2
Universite de Carthage	2

PWRI: Public Works Research Institute	2
Fed Inst Hydrol BfG	2
State University of New York (SUNY) Stony Brook	2
Sun Yat Sen University	2
Seoul National University (SNU)	2
Linkoping University	2
Kyoto University	2
North Dakota State University Fargo	2
University of Tsukuba	2
Jianghan University	2
Pusan National University	2
University System of Georgia	2
SINTEF	2
Beijing Technology & Business University	1
Guangzhou University	1
Beijing Jiaotong University	1
China Geological Survey	1
CNRS - Institute for Engineering & Systems Sciences (INSIS)	1
Guangxi Key Lab Emerging Contaminants Monitoring E	1
University of Guelph	1
University of Barcelona	1
CSIC-GV-UV - Centro de Investigaciones sobre Desertificacion (CIDE)	1
Oak Ridge Institute for Science & Education	1
CSIC - Instituto de Investigaciones Biomedicas de Barcelona (IIBB)	1
CIRAD	1
CEA	1
Technical University of Berlin	1
Universidad de Alcala	1
Bavarian Environm Agcy	1
Waters Corporation	1
Northeastern University	1
US Food & Drug Administration (FDA)	1
Danish Hydraulic Institute (DHI)	1
Krasnoyarsk Science Center of the Siberian Branch of the Russian Academy of Sciences	1
Universite de Strasbourg	1
Anhui Polytechnic University	1
Case Western Reserve University	1
National Oceanic Atmospheric Admin (NOAA) - USA	1
National Research Center for Geoanalysis	1
Anqing Normal University	1
Brno University of Technology	1
Instituto Nacional Investigacion Tecnologia Agraria Alimentaria (INIA)	1
Institute of Experimental Medicine	1
Syngenta	1

Universitat Trier	1
East China University of Science & Technology	1
Sapienza University Rome	1
Delft University of Technology	1
Catholic University of the Sacred Heart	1
Indian Institute of Technology (IIT) - Roorkee	1
Gwangju Institute of Science & Technology (GIST)	1
Agro-Environmental Protection Institute	1
University of Kragujevac	1
Concordia University - Canada	1
Connecticut Agricultural Experiment Station	1
Fed Environm Agcy Umweltbundesamt	1
Justus Liebig University Giessen	1
Eindhoven University of Technology	1
Istituto di Ricerche Farmacologiche Mario Negri IRCCS	1
Norwegian Geotechnical Institute	1
Dept Environm Sci	1
IMDEA Water Institute	1
Hebrew University of Jerusalem	1
Ecole Normale Superieure de Lyon (ENS de LYON)	1
Tsinghua University	1
Oak Ridge Associated Universities	1
Merck KGaA	1
Arizona State University	1
China Agricultural University	1
Academy of Athens	1
Finnish Environment Institute	1
University of Greenwich	1
University Hohenheim	1
Hsch Fresenius Univ Appl Sci	1
Anglia Ruskin University	1
Nevada System of Higher Education (NSHE)	1
Czech University of Life Sciences Prague	1
Ehime University	1
Dalian Institute of Chemical Physics	1
Universite de Tunis-El-Manar	1
University of Nova Gorica	1
EMD Serono Inc	1
Duke University	1
Centro de Investigaciones Energeticas	1
Bogor Agricultural University	1
Hochschule Aalen	1
Beijing Normal University	1
Graz University of Technology	1

Inst Sanit Engn Water Qual & Solid Waste Manageme	1
Arizona State University-Tempe	1
ICREA	1
CNRS - National Institute for Earth Sciences & Astronomy (INSU)	1
National Technical University of Athens	1
Carnegie Mellon University	1
Autonomous University of Barcelona	1
Bioforsk	1
Ministry of Agriculture & Rural Affairs	1
Fed Waterways Engn & Res Inst	1
Mahatma Gandhi University	1
Chinese Academy of Inspection & Quarantine	1
Chinese Center for Disease Control & Prevention	1
National Institute for Environmental Studies - Japan	1
University of Valencia	1
Hong Kong Polytechnic University	1
Deltares	1
Swedish National Food Agency	1
University of Novi Sad	1
University of Nis	1
Dept Mat & Environm Chem	1
University of Belgrade	1
Institute of Chemistry & Chemical Technology	1
Lund University	1
AgroParisTech	1
Columbia University	1
Rudjer Boskovic Institute	1
Chinese Academy of Geological Sciences	1
Universidad de Burgos	1
King's College London	1
University Osnabruck	1
Chengdu Institute of Biology	1
De Montfort University	1
University of Oslo	1
Egyptian Knowledge Bank (EKB)	1
Norwegian University of Life Sciences	1
Assiut University	1
Colorado School of Public Health	1
Colorado State University	1
Degremont	1
Brunel University	1
Public Health Agency of Barcelona	1
