# Supplementary material for: Global Trends and Hotspots in Non-Targeted Screening of Water Pollution Research: Bibliometric and Visual Analysis
Source: Toxics. 2024 Nov 24;12(12):844. doi: 10.3390/toxics12120844 (PMC11679217; doi:10.3390/toxics12120844)
Supplement: Supplementary file 1 [file toxics-12-00844-s001.zip › Supplementary table S3.pdf]

Supplementary Table S3: Display of centrality among different institutions

No.	institution	years	Centrality
1	Environment & Climate Change Canada	2016	0.21
2	Zhejiang University	2023	0.18
3	Chinese Academy of Sciences	2013	0.13
4	RWTH Aachen University	2008	0.12
5	Centre National de la Recherche Scientifique (CNRS)	2016	0.11
6	Helmholtz Association	2010	0.1
7	University of Queensland	2010	0.1
8	Nanjing University	2015	0.09
9	ETH Zurich	2010	0.07
10	Helmholtz Center for Environmental Research (UFZ)	2010	0.07
11	Consejo Superior de Investigaciones Cientificas (CSIC)	2011	0.07
12	Colorado School of Mines	2019	0.06
13	CSIC - Instituto de Diagnostico Ambiental y Estudios del Agua (IDAEA)	2012	0.05
14	Stockholm University	2012	0.05
15	CSIC - Centro de Investigacion y Desarrollo Pascual Vila (CID-CSIC)	2012	0.05
16	Eberhard Karls University of Tubingen	2012	0.05
17	Chinese Research Academy of Environmental Sciences	2022	0.05
18	China University of Geosciences	2023	0.05
19	Beijing Technology & Business University	2024	0.05
20	Guangzhou University	2024	0.05
21	Environmental Institute	2012	0.04
22	University of Luxembourg	2019	0.04
23	INRAE	2015	0.04
24	Consiglio Nazionale delle Ricerche (CNR)	2018	0.04
25	IVL Swedish Environmental Research Institute	2015	0.04
26	National & Kapodistrian University of Athens	2014	0.03
27	Universitat Jaume I	2007	0.03
28	Universidad de Almeria	2013	0.03
29	Tongji University	2023	0.03
30	University of Amsterdam	2018	0.02
31	University of Chinese Academy of Sciences	2021	0.02
32	Research Center for Eco-Environmental Sciences (RCEES)	2014	0.02
33	Technical University of Munich	2015	0.02

34	Aarhus University	2020	0.02
35	Masaryk University Brno	2015	0.02
36	NILU	2015	0.02
37	BOKU University	2016	0.02
38	Chinese Academy of Agricultural Sciences	2016	0.02
39	Institut National de l'Environnement Industriel et des Risques (INERIS)	2015	0.02
40	Peking University	2023	0.02
41	Jinan University	2023	0.02
42	Beijing Jiaotong University	2023	0.02
43	Norwegian Institute for Water Research (NIVA)	2012	0.01
44	Guangzhou Institute of Geochemistry	2022	0.01
45	Vrije Universiteit Amsterdam	2011	0.01
46	Institut Catala de Recerca de l'Aigua (ICRA)	2015	0.01
47	University of Washington	2019	0.01
48	University of Washington Tacoma	2019	0.01
49	Bureau de Recherches Geologiques et Minieres (BRGM)	2016	0.01
50	Universite Paris Cite	2021	0.01
51	Umea University	2012	0.01
52	Agilent Technologies	2018	0.01
53	South China University of Technology	2023	0.01
54	Norwegian University of Science & Technology (NTNU)	2010	0.01
55	Leibniz Institut fur Pflanzenbiochemie	2015	0.01
56	United States Environmental Protection Agency	2016	0.01
57	Universidade Estadual de Campinas	2015	0.01
