# Supplementary material for: Global Trends and Hotspots in Non-Targeted Screening of Water Pollution Research: Bibliometric and Visual Analysis
Source: Toxics. 2024 Nov 24;12(12):844. doi: 10.3390/toxics12120844 (PMC11679217; doi:10.3390/toxics12120844)
Supplement: Supplementary file 1 [file toxics-12-00844-s001.zip › Supplementary table S4.pdf]

Supplementary Table S4: Total number of publications by different journals in the field of non-targeted water pollution

Sources	Articles
SCIENCE OF THE TOTAL ENVIRONMENT	81
ENVIRONMENTAL SCIENCE & TECHNOLOGY	76
WATER RESEARCH	50
ANALYTICAL AND BIOANALYTICAL CHEMISTRY	35
JOURNAL OF HAZARDOUS MATERIALS	35
CHEMOSPHERE	30
ANALYTICAL CHEMISTRY	20
TRAC-TRENDS IN ANALYTICAL CHEMISTRY	20
ENVIRONMENTAL POLLUTION	17
ENVIRONMENTAL SCIENCES EUROPE	16
JOURNAL OF CHROMATOGRAPHY A	16
ENVIRONMENT INTERNATIONAL	11
ENVIRONMENTAL SCIENCE AND POLLUTION RESEARCH	10
ENVIRONMENTAL SCIENCE-PROCESSES & IMPACTS	10
MOLECULES	8
TALANTA	8
ENVIRONMENTAL SCIENCE-WATER RESEARCH & TECHNOLOGY	7
ANALYTICA CHIMICA ACTA	6
TRENDS IN ENVIRONMENTAL ANALYTICAL CHEMISTRY	6
WATER AIR AND SOIL POLLUTION	6
ANALYTICAL METHODS	5
ENVIRONMENTAL SCIENCE & TECHNOLOGY LETTERS	5
CHEMICAL ENGINEERING JOURNAL	4
CHINESE JOURNAL OF ANALYTICAL CHEMISTRY	4
ECOTOXICOLOGY AND ENVIRONMENTAL SAFETY	4
ENVIRONMENTAL MONITORING AND ASSESSMENT	4
ENVIRONMENTAL TOXICOLOGY AND CHEMISTRY	4
JOURNAL OF ENVIRONMENTAL CHEMICAL ENGINEERING	4
ENVIRONMENTAL RESEARCH	3
JOURNAL OF CHEMINFORMATICS	3
JOURNAL OF ENVIRONMENTAL SCIENCES	3
TOXICS	3
ABSTRACTS OF PAPERS OF THE AMERICAN CHEMICAL SOCIETY	2
CHIMIA	2
ENVIRONMENTAL CHEMISTRY LETTERS	2
ESTUARINE COASTAL AND SHELF SCIENCE	2
FOOD ADDITIVES AND CONTAMINANTS PART A-CHEMISTRY ANALYSIS CONTROL EXPOSURE & RISK ASSESSMENT	2
FOOD CHEMISTRY	2
JOURNAL OF AGRICULTURAL AND FOOD CHEMISTRY	2
JOURNAL OF MASS SPECTROMETRY	2

JOURNAL OF SOILS AND SEDIMENTS	2
JOURNAL OF THE AMERICAN SOCIETY FOR MASS SPECTROMETRY	2
JOURNAL OF WATER PROCESS ENGINEERING	2
MARINE POLLUTION BULLETIN	2
NPJ CLEAN WATER	2
RAPID COMMUNICATIONS IN MASS SPECTROMETRY	2
WATER	2
WATER SCIENCE AND TECHNOLOGY	2
ACTA HYDROCHIMICA ET HYDROBIOLOGICA	1
ANALYTICAL LETTERS	1
APPLIED CATALYSIS B-ENVIRONMENTAL	1
APPLIED MICROBIOLOGY AND BIOTECHNOLOGY	1
AQUATIC TOXICOLOGY	1
ARCHIVES OF ENVIRONMENTAL CONTAMINATION AND TOXICOLOGY	1
CANADIAN JOURNAL OF CHEMICAL ENGINEERING	1
CHEMICAL RESEARCH IN CHINESE UNIVERSITIES	1
CHEMICAL REVIEWS	1
CHEMOMETRICS AND INTELLIGENT LABORATORY SYSTEMS	1
CHINA PETROLEUM PROCESSING & PETROCHEMICAL TECHNOLOGY	1
CRITICAL REVIEWS IN ENVIRONMENTAL SCIENCE AND TECHNOLOGY	1
DESALINATION AND WATER TREATMENT	1
DRUG TESTING AND ANALYSIS	1
ENVIRONMENTAL TOXICOLOGY AND PHARMACOLOGY	1
EUROPEAN FOOD RESEARCH AND TECHNOLOGY	1
FLUID PHASE EQUILIBRIA	1
FORENSIC SCIENCE INTERNATIONAL	1
FRONTIERS IN ENVIRONMENTAL SCIENCE	1
GEOPHYSICAL RESEARCH LETTERS	1
INDUSTRIAL & ENGINEERING CHEMISTRY RESEARCH	1
INTERNATIONAL JOURNAL OF ENVIRONMENTAL RESEARCH AND PUBLIC HEALTH	1
INTERNATIONAL JOURNAL OF PHYTOREMEDIATION	1
JOURNAL OF ANALYTICAL ATOMIC SPECTROMETRY	1
JOURNAL OF CHROMATOGRAPHY B-ANALYTICAL TECHNOLOGIES IN THE BIOMEDICAL AND LIFE SCIENCES	1
JOURNAL OF CLEANER PRODUCTION	1
JOURNAL OF ENVIRONMENTAL MANAGEMENT	1
JOURNAL OF FOOD PROTECTION	1
JOURNAL OF GREAT LAKES RESEARCH	1
JOURNAL OF PHARMACEUTICAL AND BIOMEDICAL ANALYSIS	1
JOURNAL OF SEPARATION SCIENCE	1
JOURNAL OF THE BRAZILIAN CHEMICAL SOCIETY	1
JOURNAL OF THE SERBIAN CHEMICAL SOCIETY	1
JPC-JOURNAL OF PLANAR CHROMATOGRAPHY-MODERN TLC	1
LIMNOLOGICA	1

MAGNETIC RESONANCE IN CHEMISTRY	1
MASS SPECTROMETRY REVIEWS	1
MATERIALS	1
METABOLITES	1
MICROCHEMICAL JOURNAL	1
PHYTOCHEMICAL ANALYSIS	1
PHYTOMEDICINE	1
SCIENTIA HORTICULTURAE	1
